# Responding to the Humanitarian Crisis in Gaza: Damned if You do… Damned if You don’t!

**DOI:** 10.5334/aogh.3975

**Published:** 2023-08-21

**Authors:** Theresa Farhat, Sarah Ibrahim, Zahi Abdul-Sater, Ghassan Abu-Sittah

**Affiliations:** 1 Conflict Medicine Program, Global Health Institute, American University of Beirut, Beirut, Lebanon

**Keywords:** Gaza, Siege, Humanitarian, Crisis, Emergency, War, Palestine

## Abstract

Palestine, since 1948, has endured frequent military occupations and uprisings, *intifadas*, in a limited geographic area that has resulted in one of the worst humanitarian crises. The prolonged nature of this military occupation has created a biosphere of war that is uninhabitable, whereby Palestinians suffer from physical, psychological, and social wounds. Israel also imposed restrictive measures in Gaza, making it difficult for Palestinians to obtain permits to work and travel throughout Palestine. Israel continued to intensify the restrictions on Gaza, reaching a blockade on the Gaza Strip, which cut off Palestinians from Jerusalem, where hospitals, banks, and vital services are found. This form of permanent siege resulted in a surge in the unemployment rate, poverty, and poor nutritional and wellbeing status. The siege also resulted in the largest open-air prison, as people became stuck between an incomplete life and the absence of total death. The major challenge is that humanitarian interventions, in the case of Gaza, are ineffective, as they are part of the siege framework. This is because any humanitarian aid meant for Gaza needs to be approved by Israel. Thus, when the emergency becomes chronic and humanitarian interventions become part of the siege framework, how can Gaza rebuild its health capacity in a permanent emergency, and to what extent can the humanitarian sector make a change?

## Background

### History of Gaza and the siege

The Gaza Strip, now home to two million Palestinians, continuously experiences widespread destruction and human suffering and is described as “the world’s largest open-air prison” and a “laboratory” for Israel to test and refine techniques of control and management [1,2]. The concept of the Gaza Strip as a geopolitical entity is a result of Israeli settler colonial expansion and the fragmentation of Palestine. The Gaza Strip serves as a spatial representative of the settler colonial dream of Palestinian nonexistence [3]. The treatment of the Gaza Strip by Israel, including the ongoing siege and military onslaughts, demonstrates the settler’s willingness to subdue any political act or ideology that acknowledges the existence of the indigenous population [3]. Since 2007, Israel has imposed a land, air, and sea blockade on the Strip, which had and continues to have devastating effects on life in Gaza. The first war of the Israeli-Palestinian conflict and the broader Arab-Israeli conflict started in 1948. Palestinians refer to this event as *Al Nakba*, the Arabic word for “the Catastrophe,” and a signifier of a day many Palestinians fled or were expelled from their homes and became refugees in the surrounding Arab countries—initiating what is now a permanent displacement from their nation [4,5]. The *Nakba* exemplifies settler colonial invasion, where a group of settlers claims sovereignty over indigenous land, while eliminating and displacing the native majority [2]. Since that event, Palestine, especially the Gaza strip, has endured frequent wars, numerous air raids, and incursions, resulting in thousands of deaths, injuries and the displacement of people, as well as the destruction of health facilities and infrastructure [6]. The *Nakba* and its never-ending aftermath continue to define the Palestinian national identity and inform the daily lived experience of all Palestinians [5]. Today, approximately 75% of Gaza’s population is registered as refugees, most of whom rely on humanitarian aid.

## Main Text

### The uninhabitable biosphere of war

Palestine has witnessed several wars and uprisings, *intifadas*, against the military occupation, resulting in one of the worst humanitarian crises. During four major Israeli military incursions (2006 [7], 2008–09 [8], 2012 [9], and 2014 [10]), around 4,000 Palestinians were killed and over 17,000 injured [11]. Between 2018 and 2020, weekly Palestinian demonstrations were held at the border with Israel, known as the “Great Marches of Return.” These marked the 70^th^ anniversary of the *Nakba* and meant to reiterate Palestinians’ right to return to their homes and lands. These have produced a constant stream of casualties, with more than 35,000 Palestinians injured [12]. A hallmark of the Great Marches of Return was the intentional maiming of protesters by Israeli snipers, with orders to target limbs, abdomens, and pelvises. This tactic is an attempt to disable the Palestinian medical system and nation, and highlights the social suffering and far-reaching implications of injuries and amputations on Palestinians’ lives [13]. In May 2021, an 11-day war killed more than 200 Palestinians in Gaza, including over 60 children [14].

This “Warlike Condition” [15] has created a biosphere inhabited by Palestinians living in Gaza. This biosphere, characterized by the intricate interplay between living organisms and their essential natural resources, has undergone significant alterations, giving rise to multiple conduits for the injury and re-injury of the population beyond the direct initial impact of the weapons of war and the end of the shooting. The war injury in its traditional sense is no longer the blast, the shrapnel, and the gunshot wound, but is also a multitude of injuries created by this depleted and altered “biosphere of war” where the Palestinians reside [16]. The residents of Gaza endure not only physical traumas, but also psychological and social wounds linked to the combination of military occupations and the sealing of its border by Israel and its consequences (i.e., a lack of jobs, a collapsing economy, an altered living environment, a feeble health system, and lack of hope for a better life). The destruction of healthcare infrastructure in particular has added an additional layer of strain on Gaza, severely impacting the availability and accessibility of essential healthcare services for the population, further perpetuating the cycle of suffering [17].

### The violent nature of the siege

In the late 1980s, during the Palestinian uprising or *intifada*, Israel implemented restrictive measures on Palestinians, such as requiring diffficult-to-obtain permits for work and travel in occupied Palestine [1]. These restrictions were followed by the imposition of closure tactics in 1993, confining Palestinians to specific areas for months at a time [1]. The situation further deteriorated during the second *intifada* in 2000, leading to the cancellation and reduction of travel and work permits [1]. These actions by Israel are examples of structural violence, contributing to the inequalities and hardships faced by Palestinians in the Gaza Strip [18].

The most glaring form of structural violence is the 15-year-long blockade imposed on the Gaza Strip since 2007. This blockade has severed Palestinians’ access to vital services in Jerusalem, including specialized hospitals, banks, and educational opportunities. The impact of the blockade on the population is devastating, with rising unemployment rates, increased poverty levels, and a heavy reliance on international humanitarian aid for survival [19,20,21,22]. Reports indicate that the unemployment rate in the Gaza Strip is the highest in the world, doubling from 23.6% in 2005 (before the blockade) to 49% in 2020 [19]. Additionally, the poverty rate surged from 40% in 2005 to 56% in 2020, with a concurrent increase in the poverty gap from 14% to 20%. In fact, 80% of the population in Gaza rely on international humanitarian aid to survive [23]. Nutrition has also been weaponized as a result of the siege; Israel severely restrains food imports [24] and only allows a “minimal subsistence basket” that is sufficient for sustenance without the development of malnutrition [25]. As a result, about two-thirds of the people living in Gaza are food insecure today [26]. The same policy applies to other basic life essentials needed by the Palestinians in Gaza, like electricity, fuel, water, and cement, all at a severe shortage [26]. Further structural violence is committed by the siege through the restriction of the import of medicine and medical materials, as well as equipment needed for the maintenance and development of health systems—forcing hospitals and primary care centers to operate at low levels of capacity [19]. In addition, the destruction of Gaza’s infrastructure and the direct targeting of the civilian population through repeated air raids, incursions, and wars also exacerbate the deep grievances created by the siege. All this contributes to a heavier burden stretched beyond the point of collapse.

### The mechanism of titrating the siege

The siege in Palestine, exacerbated by the situation in the Gaza Strip, shares similarities with other cases of extreme economic sanctions and embargoes, such as those imposed on Iraq, Iran, and, Cuba [27,28,29]. These examples demonstrate that sieges and economic warfare rarely result in successful regime change, but instead contribute to the destruction of infrastructure and the impoverishment of the affected population. Ultimately, the siege in Palestine has created a hermetically sealed space, in which the population is trapped in an environment that hinders their ability to live a complete life while falling short of total death.

Even the humanitarian interventions in Gaza’s case, including those by international organizations and aid agencies, are part of the siege framework imposed not only by Israel but also by Egypt [30]. It is crucial to question the role of humanitarian organizations and initiatives, as many adopt a “caring for the poor” approach, rather than actively challenging the siege and advocating against it as a war crime [31]. Importantly, while these organizations provide aid, they often operate within the framework set by Israel, as their interventions require Israel’s approval, effectively maintaining the status quo of the continuous siege. Israel’s orchestration of humanitarian aid further reveals the plight faced by these organizations, as they find themselves in a dilemma of being instrumentalized by the siege by providing conditional aid or causing catastrophic consequences by halting aid. This was evident even before the *Nakba*. The United Nations Relief and Works Agency for Palestine Refugees in the Near East (UNRWA) was established to address the immediate needs of refugees until a lasting solution could be found. Over time, it has become increasingly interconnected with the long-standing Palestinian refugee crisis. More recently, this was evident when international humanitarian teams were not allowed to provide any aid or services to Gaza for a period of time, until the war in 2014 [30]. The humanitarian organizations find themselves in a situation where they are damned if they do, and they are damned if they don’t. The paradoxical state of humanitarian organizations and their role in maintaining the status quo for Palestinians have been there since the *Nakba*, with the UNRWA, refugee relief foundations, and various agencies as examples. Israel also has a responsibility to uphold law and order, or “normal life,” in the area it occupies. In accordance with international law, especially Occupation Law, which describes Israel’s obligations as an occupying power, this commitment includes maintaining the safety and welfare of the occupied population [32].

### The “forever emergency” and challenges of responding

“It’s layer-upon-layer of crisis, and there never is enough time between each crisis to rebuild,” said Matthias Schmale, the UNRWA director in Gaza, and therefore with every new battle, the same challenges re-emerge. The wars and the siege are not only a consequence of the political situation, but they are also a weapon of the political situation that aims to prevent the reconstruction of what was destroyed; it is a policy rather than a consequence of the chaos of the war. This “forever emergency” is a consequence of the long-lasting blockade and closure of Gaza and Israel’s military occupations, resulting in a protracted human catastrophe. The forever emergency, which began in 1948 at the beginning of the Arab-Israeli War, has turned an already dire situation into a humanitarian catastrophe and has cultivated widespread hopelessness among Gaza’s population [33]. The resulting high dependence (80% of the population) on food subsidies from international organizations, severe poverty, malnutrition, and a lack of vital supplies (such as water and electricity) have jointly made Gaza uninhabitable and a catastrophe [34].

## Conclusion

When an emergency becomes chronic, and when humanitarian interventions become part of the grand design of the inhumane, unrelenting siege and a part of the titration mechanism, important questions arise: how does the humanitarian sector respond? Does its duty to bear witness overrule its duty to provide a service? Another important question is how Gaza can build its health capacity, when it is continuously trying to respond to emergencies. It is important to promote comprehensive solutions to reduce the suffering of Palestinians while simultaneously posing these issues. These include ending the siege and blockade, giving aid organizations the freedom to carry out their work, and creating an atmosphere that fosters the expansion of Gaza’s healthcare infrastructure.
